# Epidemiology of Posterior Heel Pain in the General Population: Cross‐Sectional Findings From the Clinical Assessment Study of the Foot

**DOI:** 10.1002/acr.22546

**Published:** 2015-06-25

**Authors:** Benjamin D. Chatterton, Sara Muller, Edward Roddy

**Affiliations:** ^1^ Research Institute for Primary Care and Health Sciences, Keele UniversityStaffordshireUK

## Abstract

**Objective:**

To identify the population prevalence of posterior heel pain (HP), related disability, and associated factors.

**Methods:**

A total of 9,334 adults ages ≥50 years were mailed a questionnaire. Participants reporting foot pain in the last month shaded the foot pain location on a manikin. The Manchester Foot Pain and Disability Index assessed disabling foot pain. Population prevalence of any, bilateral, and disabling posterior HP was estimated using weighted logistic regression accounting for nonresponse. Odds ratios (ORs) and 95% confidence intervals (95% CIs) were calculated between posterior HP and age, sex, neighborhood deprivation, occupational class (professional, intermediate, and manual), body mass index (BMI, kg/m^2^), physical activity, heel height, and diabetes mellitus.

**Results:**

A total of 5,109 questionnaires were received (adjusted response 56%). Six hundred seventy‐five respondents (13%) reported posterior HP, of whom 382 had bilateral symptoms. A total of 398 (8%) reported disabling posterior HP. Posterior HP in either foot was associated with increasing BMI (25.0–29.9 [OR 1.58], 30.0–34.9 [OR 2.13], and ≥35.0 [OR 4.09]) and with manual occupations (OR 1.96, 95% CI 1.47–2.62). Bilateral posterior HP was associated with increasing BMI (25.0–29.9 [OR 1.79], 30.0–34.9 [OR 2.43], and ≥35.0 [OR 5.79]), diabetes mellitus (OR 1.48, 95% CI 1.07–2.05), and manual occupations (OR 2.21, 95% CI 1.48–3.30). Disabling posterior HP was associated with increasing BMI (25.0–29.9 [OR 1.44], 30.0–34.9 [OR 2.50], and ≥35.0 [OR 4.69]), age (≥75 years OR 1.41, 95% CI 1.01–1.96), manual occupations (OR 1.97, 95% CI 1.35–2.88), and diabetes mellitus (OR 1.56, 95% CI 1.04–1.95). High physical activity was negatively associated with posterior HP in either heel (OR 0.43, 95% CI 0.33–0.56), bilateral posterior HP (OR 0.35, 95% CI 0.25–0.49), and disabling posterior HP (OR 0.33, 95% CI 0.23–0.46).

**Conclusion:**

Posterior HP is prevalent and associated with obesity, manual occupations, and physical inactivity. Prospective studies should assess the roles of obesity in causation and weight loss in treatment.

## INTRODUCTION

Foot pain is a common symptom in the general population, particularly in older adults. The prevalence in adults ages ≥18 years ranges from 17–24% 1, 2, rising to as high as 42% in adults ages >65 years 3. Three‐quarters of older adults with foot pain experience disabling pain affecting them on most days 4, and foot pain is also linked to problems with mobility and gait in this age group, with an increased risk of falling 5, 6.

Box 1 **Significance & Innovations**

- This is the first large population‐based study to report the prevalence of foot pain specific to the posterior heel.
- Posterior heel pain (HP) affects 1in 8 of the population ages ≥50 years, and over half of these report disabling foot pain.
- Posterior HP is significantly associated with obesity, routine and manual occupations, and low physical activity levels.

Although the prevalence of foot pain has been well reported, the contribution of the posterior heel to this prevalence is less clear. Previous studies have reported pain by area of the foot 1, 2, but in these the heel is reported as a single region rather than by precise anatomic site. Pain in specific anatomic areas of the foot is often attributed clinically to specific conditions, and therefore observing pain at a specific site can give greater insights into the underlying pathology.

The etiology of posterior heel pain (HP) is largely related to disorders of the Achilles tendon and associated structures and varies by age and location. In children and adolescents, the most common cause is calcaneal apophysitis (Sever's disease) 7. In adults, midportion posterior HP is likely to represent Achilles tendinopathy, whereas insertional pain may occur due to Haglund's deformity, retrocalcaneal bursitis, or enthesitis 7, 8.

Previous studies have reported the incidence of specific posterior heel pathologies, but the population prevalence of posterior HP is unknown. De Jonge et al note an incidence rate of Achilles tendinopathy in Dutch general practice consulters of 1.85 per 1,000 9. Overuse, sporting injuries, obesity, and diabetes mellitus have previously been implicated in the pathogenesis of Achilles tendon disorders 10, 11, 12, 13. However, these associations have largely been reported from retrospective case reviews in specialist settings or small cohort studies in specific populations (for example, elite athletes) 10. Further information on the overall population prevalence and risk factors for developing posterior HP would inform health service provision and identify potential preventative and treatment strategies.

The aims of this study were therefore to estimate the population prevalence of posterior HP in either foot, bilateral posterior HP, and disabling posterior HP in community‐dwelling adults ages ≥50 years, and to examine factors that may be associated with posterior HP.

## PATIENTS AND METHODS

#### Study design

Baseline data were obtained from the Clinical Assessment Study of the Foot, a 3‐year prospective observational population‐based cohort study 14. In brief, a baseline postal Health Survey questionnaire was sent to all adults ages ≥50 years registered at 4 general practices in the North Staffordshire region of England, UK, irrespective of consultation for foot pain. The survey was accompanied by a letter of invitation from the participant's general practitioner and an information leaflet. Consent to participate was implied by return of the questionnaire. Ethical approval was obtained from the Coventry Research Ethics Committee (reference number 10/H1210/5).

#### Data collection

##### Posterior HP

**Figure 1:**
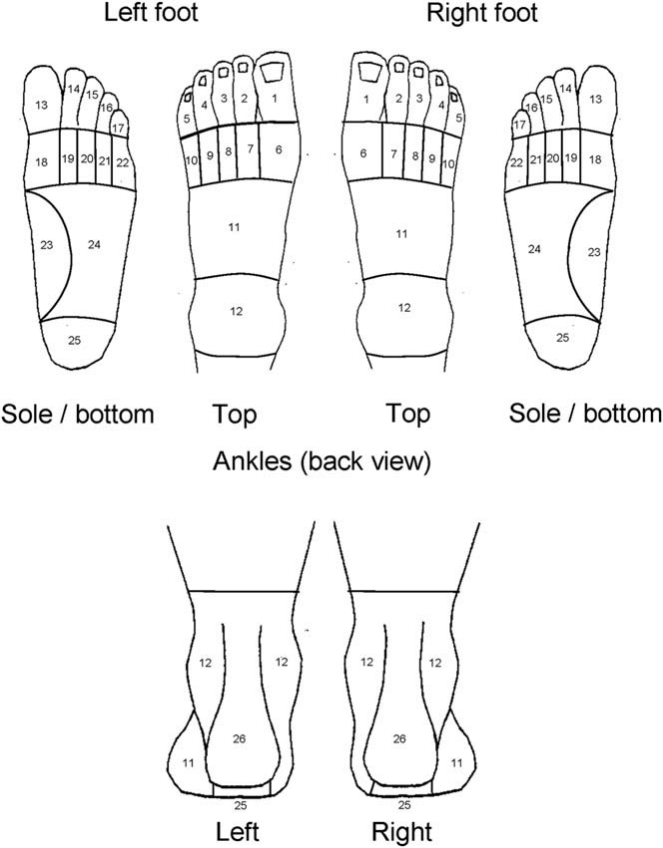
The foot pain manikin scoring areas used in the Health Survey questionnaire. Diagram originally created by Dr. Adam Garrow at The University of Manchester. Reproduced by permission (The University of Manchester).

The Health Survey questionnaire contained the filter question “In the past month, have you had any ache or pain that has lasted for one day or longer in your feet?” Respondents reporting foot ache or pain in the past month were asked to shade the location of their foot ache/pain on a foot manikin showing the dorsal, plantar, and posterior aspects of both feet 2. The manikins were scored with a transparent overlay, dividing the images of the feet into 26 mutually exclusive areas 15. Any area with shading was considered to be painful, and any area with no shading not to be painful. Posterior HP in either foot was defined as reporting foot ache or pain in the past month and shading the posterior heel (area 26) on either foot 15. Bilateral posterior HP was defined as responding positively to the filter question and shading the posterior heel (area 26) in both feet (Figure 1).

##### Disabling posterior HP

Disabling foot pain was assessed using the 10‐item function construct of the Manchester Foot Pain and Disability Index (MFPDI) 16. The function construct includes statements such as “Because of pain in my feet, I walk slowly,” with respondents asked to score each item as occurring “none of the time,” “on some days,” or “on most/every day(s).” The presence of disabling posterior HP was defined as reporting at least 1 of the 10 function items in the MFPDI function construct as occurring “on most/every day(s)” 4, together with the presence of posterior HP in either foot in the past month, as defined above.

##### Associations of posterior HP

The association of posterior HP with the following factors was assessed: age, sex, body mass index (BMI, kg/m^2^), individual occupational class, neighborhood deprivation quartile (NDQ), physical activity level, heel height, and diabetes mellitus. Age was categorized as 50–64 years, 65–74 years, and ≥75 years. BMI was calculated from self‐reported height and weight, and categorized as <25.0, 25.0–29.9, 30.0–34.9, or ≥35.0. Sex and diabetes mellitus were self‐reported in the Health Survey questionnaire. Respondents were asked to report their current or most recent job title. Individual occupational class was then ranked using the UK Office for National Statistics 3‐tier socioeconomic classification: 1) higher managerial, administrative, and professional occupations, 2) intermediate occupations, or 3) routine and manual occupations 17. A fourth category, “other,” was used to encompass housewives, nonworkers, retired people, and inadequately described responses, and was excluded from analysis. The NDQ was calculated from the respondent's Index of Deprivation score, derived from the respondent's post code using UK Government Department for Communities and Local Government data. The indices of deprivation are used as a relative measure of deprivation for an area in England, and are generated from 38 indicators, grouped into 7 domains that represent different aspects of deprivation. These include income, employment, health, education, crime, access to services, and living environment. An area is assigned a deprivation score from these domains, with deprivation decreasing with an increasing score, and respondents were categorized into quartiles using this score for their area 18. Physical activity was assessed using the short form International Physical Activity Questionnaire, a validated 4‐item construct that assesses physical activity levels over the previous 7 days 19. The construct contains questions such as “During the last 7 days, on how many days did you walk for at least 10 minutes at a time?” Respondents were categorized into tertiles corresponding to low, medium, and high physical activity groups. Heel height use was assessed in female respondents using shoe diagrams depicting 4 different heel heights 14. Respondents were asked to indicate which picture showed the height of the heel they wore most of the time in different decades of their life. High‐heel exposure was defined as a participant selecting either of the pictures depicting the 2 highest heels as that worn on most days for at least one 10‐year period between 20 and 49 years.

#### Statistical analysis

Demographic characteristics of the study population were defined using descriptive statistics. The prevalence of posterior HP in either foot, bilateral posterior HP, and disabling posterior HP were first calculated as a percentage of the responder population. These estimates were then weighted to account for selective nonresponse from the eligible mailed baseline population. Using information on age, sex, and general practice available for both responders and nonresponders, weighted logistic regression was used to determine prevalence estimates and 95% confidence intervals (95% CIs) in the eligible baseline mailed population.

Univariate logistic regression modeling was used to calculate odds ratios (ORs) and 95% CIs between posterior HP and age, sex, BMI, NDQ, individual occupational class, physical activity level, and diabetes mellitus. A multivariate model was then performed that included age, sex, BMI, occupational class, NDQ, physical activity level, and diabetes mellitus, generating fully adjusted ORs for associations between any posterior HP, bilateral posterior HP, and disabling posterior HP with the above factors. Separate univariate and multivariate models for posterior HP, bilateral posterior HP, and disabling posterior HP were then performed in women only, including heel height as an additional variable. All data analyses were performed using SPSS Statistics for Windows, version 20.0, with the exception of prevalence values weighted for nonresponders, which were calculated using Stata Statistical Software, version 12.

## RESULTS

#### Study population

The baseline Health Survey questionnaire was initially sent to 9,334 adults ages ≥50 years. During the mailing process, there were 140 exclusions due to deaths, ill health, departures, and incorrect addresses. The exclusions left an eligible baseline mailed population of 9,194 people, from whom 5,109 completed Health Survey questionnaires were received (adjusted response 56%). As reported previously, the age, sex, and practice distribution of responders were broadly representative of the baseline eligible population 20. Forty‐eight percent of the study population were men, with the majority of respondents ages 50–64 years (50%). Further demographic characteristics of the responder population are shown in Table 1.

**Table 1:** Demographic characteristics of baseline Health Survey responders^a^

	Responders (n = 5,109)
Sex	
Men	2,439 (48)
Women	2,670 (52)
Age, years	
50–64	2,563 (50)
65–74	1,530 (30)
≥75	1,016 (20)
Neighborhood deprivation (quartiles)	
Least deprived	1,143 (21)
Upper mid‐deprived	1,405 (28)
Lower mid‐deprived	1,259 (25)
Most deprived	1,302 (26)
Individual occupational class	
Higher managerial/administrative/professional	1,011 (20)
Intermediate	887 (17)
Routine and manual	2,668 (52)
Other^b^	543 (11)
Body mass index, kg/m^2^	
<25.0	1,684 (33)
25.0–29.9	1,948 (38)
30.0–34.9	840 (16)
≥35.0	389 (8)
Missing	248 (5)
Physical activity level	
Low	1,119 (22)
Medium	1,820 (36)
High	1,393 (27)
Missing	777 (15)
Heel height (women only)	
Low heel	808 (30)
High heel	1,402 (53)
Missing	460 (17)
Diabetes mellitus	693 (14)

a Values are the number (%).

b Includes housewives, nonworkers, retired people, and those inadequately described.

#### Population prevalence of posterior HP

Of 5,109 respondents, 675 (13.2%) reported posterior HP in either foot. Of these, 382 (7.5%) had bilateral posterior HP, and 398 (8%) disabling posterior HP. After weighting back to the mailed population, the population prevalence in adults ages ≥50 years was estimated to be 13.4% for posterior HP in either foot (95% CI 12.5–14.4), 7.6% for bilateral posterior HP (95% CI 6.9–8.3), and 7.9% for disabling posterior HP (95% CI 7.2–8.7). Of the 398 respondents reporting disabling posterior HP, 391 (98.2%) also reported pain in other foot areas.

#### Factors associated with posterior HP in either foot

Having posterior HP in either foot was significantly associated with a higher BMI, with a dose‐response relationship observed. Compared to those with a BMI <25.0, the adjusted OR for posterior HP in either foot was 1.58 (95% CI 1.22–2.04) in those with a BMI 25.0–29.9, 2.13 (95% CI 1.58–2.85) in those with a BMI 30.0–34.9, and 4.09 (95% CI 2.90–5.75) in those with a BMI ≥35.0. A significant association was also seen with routine and manual occupations (adjusted OR 1.96, 95% CI 1.47–2.62) when compared to professional occupations. Those with higher levels of physical activity were less likely to have posterior HP when compared to those with low activity levels (medium activity levels adjusted OR 0.58, 95% CI 0.46–0.73; high activity levels 0.43, 95% CI 0.33–0.56). Significant associations were seen on univariate analysis for female sex, deprivation, and diabetes mellitus, but these associations were not significant on multivariate analysis (Table 2). Heel height (univariate OR 1.26, 95% CI 0.98–1.63; adjusted OR 1.03, 95% CI 0.76–1.40) and age showed no association with pain in either heel.

**Table 2:** Factors associated with posterior heel pain in either foot^a^

	**Posterior heel pain, no. (%)**	**No posterior heel pain, no. (%)**	**Unadjusted OR (95% CI)**	**Adjusted OR (95% CI)** b
Sex				
Men	290 (43.0)	2,149 (48.5)	1.0	1.0
Women	385 (57.0)	2,285 (51.5)	1.25 (1.06–1.47)	1.13 (0.93–1.39)
Age, years				
50–64	343 (50.8)	2,220 (50.1)	1.0	1.0
65–74	190 (28.1)	1,340 (30.2)	0.92 (0.76–1.11)	0.89 (0.71–1.12)
≥75	142 (21.0)	874 (19.7)	1.05 (0.85–1.30)	1.01 (0.77–1.34)
Body mass index, kg/m^2^				
<25.0	145 (22.6)	1,539 (36.5)	1.0	1.0
25.0–29.9	243 (37.8)	1,705 (40.4)	1.51 (1.22–1.88)	1.58 (1.22–2.04)
30.0–34.9	139 (21.6)	701 (16.6)	2.11 (1.64–2.70)	2.13 (1.58–2.85)
≥35.0	116 (18.0)	273 (6.5)	4.51 (3.42–5.94)	4.09 (2.90–5.75)
Neighborhood deprivation (quartiles)				
Least deprived	116 (17.2)	1,027 (23.2)	1.0	1.0
Upper mid‐deprived	165 (24.4)	1,240 (28.0)	1.17 (0.92–1.52)	1.13 (0.83–1.53)
Lower mid‐deprived	187 (27.7)	1,072 (24.2)	1.54 (1.20–1.98)	1.12 (0.82–1.50)
Most deprived	207 (30.7)	1,095 (24.7)	1.67 (1.31–2.13)	0.98 (0.72–1.32)
Individual occupational class				
Higher managerial/administrative/professional	84 (14.2)	927 (23.3)	1.0	1.0
Intermediate	91 (15.4)	796 (20.0)	1.26 (0.92–1.72)	1.28 (0.91–1.81)
Routine and manual	416 (70.4)	2,252 (56.7)	2.04 (1.59–2.61)	1.96 (1.47–2.62)
Physical activity level				
Low	228 (40.2)	891 (23.7)	1.0	1.0
Medium	218 (38.4)	1,602 (42.5)	0.53 (0.43–0.65)	0.58 (0.46–0.73)
High	121 (21.3)	1,272 (33.8)	0.37 (0.29–0.47)	0.43 (0.33–0.56)
Diabetes mellitus				
Absent	545 (80.7)	3,871 (87.3)	1.0	1.0
Present	130 (19.3)	563 (12.7)	1.64 (1.33–2.03)	1.29 (0.98–1.69)

a OR = odds ratio; 95% CI = 95% confidence interval.

b OR adjusted for age, sex, body mass index, occupational class, neighborhood deprivation, physical activity, and presence of diabetes mellitus.

#### Factors associated with bilateral posterior HP

A similar dose‐response relationship was seen between bilateral posterior HP and higher BMI (kg/m^2^). Compared to those with a BMI <25.0, the adjusted OR was 1.79 (95% CI 1.25–2.57) in those with a BMI 25.0–29.9, 2.43 (95% CI 1.63–3.62) in those with a BMI 30.0–34.9, and 5.79 (95% CI 3.77–8.89) in those with a BMI ≥35.0. As with pain in either heel, bilateral posterior HP was significantly less common in those with high activity levels. When compared to those with low activity, the adjusted OR for medium activity was 0.48 (95% CI 0.35–0.64), and 0.35 for high activity (95% CI 0.25–0.49). Bilateral pain was again significantly associated with female sex and deprivation in univariate modeling, but these associations were not significant on multivariate analysis (Table 3). Diabetes mellitus was, however, significantly associated with bilateral posterior HP on multivariate analysis (OR 1.48, 95% CI 1.07–2.05), as was having held a routine and manual occupation (OR 2.21, 95% CI 1.48–3.30). Heel height was significantly associated with bilateral pain in univariate modeling in women only (OR 1.44, 95% CI 1.03–2.02), but this association was not reproduced in multivariate modeling (adjusted OR 1.04, 95% CI 0.70–1.55). As with pain in either heel, no association was seen with increasing age.

**Table 3:** Factors associated with bilateral posterior heel pain^a^

	**Posterior heel pain, no. (%)**	**No posterior heel pain, no. (%)**	**Unadjusted OR (95% CI)**	**Adjusted OR (95% CI)** b
Sex				
Men	161 (42.1)	2,278 (48.2)	1.0	1.0
Women	221 (57.9)	2,449 (51.8)	1.28 (1.03–1.58)	1.17 (0.90–1.52)
Age, years				
50–64	190 (49.7)	2,373 (50.2)	1.0	1.0
65–74	105 (27.5)	1,425 (30.1)	0.92 (0.72–1.18)	0.85 (0.63–1.16)
≥75	87 (22.8)	929 (19.7)	1.17 (0.90–1.52)	1.18 (0.83–1.66)
Body mass index, kg/m^2^				
<25.0	70 (19.4)	1,614 (35.9)	1.0	1.0
25.0–29.9	134 (37.2)	1,814 (40.3)	1.70 (1.27–2.29)	1.79 (1.25–2.57)
30.0–34.9	73 (20.3)	767 (17.0)	2.19 (1.56–3.08)	2.43 (1.63–3.62)
≥35.0	83 (23.1)	306 (6.8)	6.25 (4.45–8.79)	5.79 (3.77–8.89)
Neighborhood deprivation (quartiles)				
Least deprived	57 (14.9)	1,086 (23.0)	1.0	1.0
Upper mid‐deprived	95 (24.9)	1,310 (27.7)	1.38 (0.99–1.94)	1.33 (0.87–2.02)
Lower mid‐deprived	110 (28.8)	1,149 (24.3)	1.82 (1.31–2.54)	1.39 (0.92–2.11)
Most deprived	120 (31.4)	1,182 (25.0)	1.93 (1.40–2.68)	1.19 (0.78–1.80)
Individual occupational class				
Higher managerial/administrative/professional	43 (12.6)	968 (22.9)	1.0	1.0
Intermediate	52 (15.3)	835 (19.8)	1.40 (0.93–2.12)	1.47 (0.91–2.36)
Routine and manual	245 (72.1)	2,423 (57.3)	2.28 (1.63–3.17)	2.21 (1.48–3.30)
Physical activity level				
Low	147 (45.8)	972 (24.2)	1.0	1.0
Medium	114 (35.5)	1,706 (42.5)	0.44 (0.34–0.57)	0.48 (0.35–0.64)
High	60 (18.7)	1,333 (33.2)	0.30 (0.22–0.41)	0.35 (0.25–0.49)
Diabetes mellitus				
Absent	293 (76.7)	4,123 (87.2)	1.0	1.0
Present	89 (23.3)	604 (12.8)	2.07 (1.61–2.67)	1.48 (1.07–2.05)

a OR = odds ratio; 95% CI = 95% confidence interval.

b OR adjusted for age, sex, body mass index, occupational class, neighborhood deprivation, physical activity, and presence of diabetes mellitus.

#### Factors associated with disabling posterior HP

A significant association between disabling posterior HP and BMI was present on multivariate modeling. Compared to those with a BMI <25.0, the adjusted OR was 1.44 (95% CI 1.02–2.02) in those with a BMI 25.0–29.9, OR 2.50 (95% CI 1.73–3.59) in those with a BMI 30.0–34.9, and OR 4.69 (95% CI 3.12–7.08) in those with a BMI ≥35.0.

Those with higher activity levels were significantly less likely to experience disabling posterior HP. When compared to low activity, the adjusted OR for medium activity was 0.53 (95% CI 0.40–0.69), and for high activity 0.33 (95% CI 0.23–0.46). Significant associations were also seen with ages ≥75 years (adjusted OR 1.41, 95% CI 1.01–1.96), intermediate occupational class (adjusted OR 1.86, 95% CI 1.21–2.85), routine and manual occupational class (adjusted OR 1.97, 95% CI 1.35–2.88), and diabetes mellitus (adjusted OR 1.56, 95% CI 1.04–1.95) (Table 4). Significant associations for disabling posterior HP were seen in univariate modeling with deprivation and having worn high heels previously in women only (univariate OR 1.40, 95% CI 1.02–1.94 and adjusted OR 1.13, 95% CI 0.78–1.66), but these associations were no longer significant on multivariate analysis (Table 4).

**Table 4:** Factors associated with disabling foot pain in those with posterior heel pain^a^

	**Disabling pain, no. (%)**	**No disabling pain, no. (%)**	**Unadjusted OR (95% CI)**	**Adjusted OR (95% CI)** b
Sex				
Men	171 (43.0)	54 (50.9)	1.0	1.0
Women	227 (57.0)	52 (49.1)	1.38 (0.90–2.12)	1.14 (0.89–1.47)
Age, years				
50–64	197 (49.5)	74 (69.8)	1.0	1.0
65–74	111 (27.9)	28 (26.4)	1.49 (0.91–2.44)	1.08 (0.81–1.44)
≥75	90 (22.6)	4 (3.8)	8.45 (2.30–23.83)	1.41 (1.01–1.96)
Body mass index, kg/m^2^				
<25.0	81 (21.1)	26 (25.2)	1.0	1.0
25.0–29.9	129 (33.7)	47 (45.6)	0.88 (0.51–1.53)	1.44 (1.02–2.02)
30.0–34.9	89 (23.2)	19 (18.4)	1.50 (0.77–2.92)	2.50 (1.73–3.59)
≥35.0	84 (21.9)	11 (10.7)	2.45 (1.14–5.28)	4.69 (3.12–7.08)
Neighborhood deprivation (quartiles)				
Least deprived	65 (16.3)	29 (27.4)	1.0	1.0
Upper mid‐deprived	101 (25.4)	25 (23.6)	1.80 (0.97–3.35)	1.22 (0.82–1.80)
Lower mid‐deprived	106 (26.6)	31 (29.2)	1.53 (0.84–2.76)	1.11 (0.75–1.65)
Most deprived	126 (31.7)	21 (19.8)	2.68 (1.42–5.06)	1.14 (0.78–1.67)
Individual occupational class				
Higher managerial/administrative/professional	45 (12.7)	19 (18.4)	1.0	1.0
Intermediate	72 (20.4)	9 (8.7)	3.38 (1.41–8.11)	1.86 (1.21–2.85)
Routine and manual	236 (66.9)	75 (72.8)	1.33 (0.73–2.41)	1.97 (1.35–2.88)
Physical activity level				
Low	158 (46.2)	961 (24.1)	1.0	1.0
Medium	126 (36.8)	1,694 (42.5)	0.45 (0.35–0.58)	0.53 (0.40–0.69)
High	58 (17.0)	1,335 (33.5)	0.26 (0.19–0.36)	0.33 (0.23–0.46)
Diabetes mellitus				
Absent	310 (77.9)	268 (96.8)	1.0	1.0
Present	88 (22.1)	9 (3.2)	3.06 (1.49–6.30)	1.5643 (1.04–1.95)

a OR = odds ratio; 95% CI = 95% confidence interval.

b OR adjusted for age, sex, body mass index, occupational class, neighborhood deprivation, physical activity, and presence of diabetes mellitus.

## DISCUSSION

To our knowledge, this is the first large population‐based study to report the prevalence of foot pain specific to the posterior heel. Our findings suggest that posterior HP is a common problem for adults ages ≥50 years, affecting just over 1 in 8 of the general population. In addition, over half of those with posterior HP were disabled by their pain. Obesity and routine and manual occupations were associated with posterior HP in either heel; bilateral posterior HP was additionally associated with diabetes mellitus, and disabling pain was associated with increasing age. In addition, those with medium and high physical activity levels were significantly less likely to experience pain in either heel, bilateral pain, and disabling pain.

Previous studies have reported prevalence of pain in the broader anatomic region of the hindfoot, derived from the foot manikin used in the Framingham Foot Study 1, 21. Using this manikin in the North West Adelaide Health Study, Hill et al reported a prevalence of hindfoot pain that ranged from 21.0% in adults ages 45–54 years to 32.8% in those ages ≥75 years 1. Dufour et al reported a much lower prevalence of hindfoot pain of 8% using the Framingham manikin 21. Our prevalence estimate is likely lower than that of Hill et al because their definition of hind‐foot pain covered a larger anatomic region than our definition of posterior HP, and our prevalence estimate may be higher than that reported by Dufour et al because their population included younger adults than our population. Disability related to foot pain has also been well reported, with 10–64% of those with foot pain reporting disability 2, 4.

Perhaps the most striking association of posterior HP was seen with obesity, as those having a higher BMI were increasingly likely both to experience posterior HP and to be disabled by it. These findings are in keeping with previous retrospective case–control studies undertaken in specialist settings, which found that those with Achilles tendinopathy were 2.6 to 6.6 times more likely to be obese 11, 12. The association of diabetes mellitus with posterior HP has also been suggested by previous literature. In animal models, the Achilles tendons of rats with induced diabetes mellitus demonstrate significant structural and inflammatory changes 22. The Achilles tendons of asymptomatic diabetic individuals show increased sonographic structural abnormalities when compared to controls 13, and patients with diabetes mellitus are known to have an increased incidence of other tendon disorders, including symptomatic and asymptomatic tears of the rotator cuff and biceps tendon 23.

An association was also found with age and disabling posterior HP on multivariate analysis. These findings are in keeping with a previous large epidemiologic study reporting that although pain prevalence did not increase with age in those ages ≥50 years, the extent to which pain interferes with everyday life did 24. The association of posterior HP with low occupational class would also be in keeping with previous literature. The prevalence of symptomatic radiographic foot osteoarthritis is increased in lower socioeconomic classes 19, and those with higher neighborhood deprivation are more likely to experience severe pain and be unable to work as a consequence 25. Recently, Lacey et al have also reported that those whose longest job during their lifetime has been in the routine and manual occupational class are more likely to experience disabling pain 26.

The association of foot pain, and in particular heel pain, with activity levels has been less well studied, with previous research focusing on associations with the sequelae of inactivity, such as obesity and diabetes mellitus. However, Rano et al noted in a case–control study of patients with heel pain that the controls reported higher activity levels than patients with heel pain 27. With regard to the effect of heel height on posterior HP, there is again a relatively small body of evidence. The lack of association in multivariate modeling between posterior HP and previous wearing of high heels may be explained by the biomechanic changes in the foot when high heels are worn. Specifically, several studies have noted that when high heels are worn, plantar pressure shifts from the heel and Achilles tendon to the medial forefoot 28, 29. It is also possible the findings relating to heel height were influenced by recall bias arising from an exposure definition ascertained using a retrospective questionnaire.

The strengths of this study include a large population size, the community setting, and the use of a detailed, previously validated foot manikin that allows accurate characterization of pain location 15. The use of weighted logistic regression modeling has also allowed us to adjust for nonresponse to the initial survey and to account for confounding variables in our assessment of associations for posterior HP. There are, however, limitations to this study that warrant consideration. The response to the postal survey was moderate, although the study population was broadly representative of the mailed population, as reported previously 20. Participants did not undergo clinical assessment of the posterior heel, so we were not able to assess the cause for their pain and have made an assumption that it is largely attributable to Achilles tendon disorders. A further caveat is that questions about foot‐related disability were asked in reference to foot pain in general, rather than posterior HP specifically, and in addition the majority of those with disabling pain had shaded more than 1 area on the foot manikin (98.2%), meaning we are unable to say categorically that foot‐related disability is occurring as a result of posterior HP. Finally, because the study was cross‐sectional, we are unable to determine temporal aspects of these associations or causality.

The main clinical implications of this study arise from our findings that posterior HP affects 1 in 8 of the population age ≥50 years and is associated with significant functional limitation, particularly in the obese, older people, those with diabetes mellitus, and those in routine and manual occupations, and it is significantly less common in those with high activity levels. This finding suggests that posterior HP is a significant public health issue, and with an aging population and an increasing prevalence of obesity and diabetes mellitus, the likelihood is that the prevalence of posterior HP will also continue to rise. Further research is needed into the causality of posterior HP and in particular its associations with obesity, diabetes mellitus, and activity. This causality could highlight potential preventative measures, particularly given the fact that those with higher activity levels are less likely to experience posterior HP. The role of weight loss and glycemic control as treatment strategies in managing posterior HP could also be explored.

## AUTHOR CONTRIBUTIONS

All authors were involved in drafting the article or revising it critically for important intellectual content, and all authors approved the final version to be submitted for publication. Dr. Chatterton had full access to all of the data in the study and takes responsibility for the integrity of the data and the accuracy of the data analysis.

**Study conception and design.** Chatterton, Muller, Roddy.

**Acquisition of data.** Roddy.

**Analysis and interpretation of data.** Chatterton, Muller, Roddy.
